# Millimeter accuracy depth estimation in concrete using smartphone-equipped handheld GPR and hyperbolic curve fitting with antenna distance consideration

**DOI:** 10.1016/j.heliyon.2024.e38154

**Published:** 2024-09-24

**Authors:** Shunsuke Iwai, Tsukasa Mizutani

**Affiliations:** Institute of Industrial Science, The University of Tokyo, 4-6-1, Koamaba, Meguro-ku, Tokyo, 153-8506, Japan

**Keywords:** Smartphone-equipped handheld GPR, Concrete, Depth estimation, Hyperbolic curve fitting, Inter-antenna distance

## Abstract

In the management and maintenance of structural facilities, knowing the cover thickness of rebar and the depth of buried objects is of paramount importance. Currently, Smartphone-Equipped Handheld Ground Penetrating Radars (GPR) are primarily used for detecting rebar, yet the Electromagnetic Induction (EMI) method remains the sole available technique for determining the cover thickness of rebar. This necessitates the use of two separate devices to understand the subsurface condition of rebar. In this study, we introduce a new algorithm that utilizes Hyperbolic Curve Fitting to determine depth and relative permittivity from reflection images derived from a single GPR measurement, using metal depth estimation as an exemplar. Our contributions include the automatic detection of the hyperbola's centerline before curve fitting, separation of the hyperbola into left and right for enhanced fitting accuracy by removing points near the vertex, and incorporating a rigorous theoretical model that took into account the distance between the transmitting and receiving antennas, known as the inter-antenna distance. This algorithm had enabled us to meet the performance requirements for the EMI method as stipulated by the Japanese Society for Non-Destructive Inspection (JSNDI). Notably, our method retained its accuracy even at depths exceeding the 100 mm limitation of the EMI method.

## Highlights

• This study proposes a new method to determine depth and relative permittivity using a single GPR measurement.
• By focusing on the symmetry of the hyperbola, we were able to automatically determine the fitting range and the centerline of the hyperbola.
• We developed an algorithm unaffected by object size by splitting the hyperbola and excluding the vertex area.
• Using a corrected inter-antenna distance model, we accurately estimated the rebar depth with a maximum error of 2.67%.

## 1 Introduction; depth estimation in concrete using ground penetrating radar signals

Ground Penetrating Radar (GPR) is frequently employed as a non-destructive inspection (NDI) tool in infrastructure maintenance due to its capacity to visualize invisible subsurface of the structures and its high operational efficiency [1]. GPR devices vary significantly in size, ranging from small handheld units to larger hand-pushed and vehicle-mounted systems. GPR devices serve diverse functions such as rebar inspection [2], [3], [4], [5], [6], slab thickness estimation [7], [8], [9], [10], [11], [12], [13], [14], [15], [16], [17], [18], [19].

**Figure 1:**
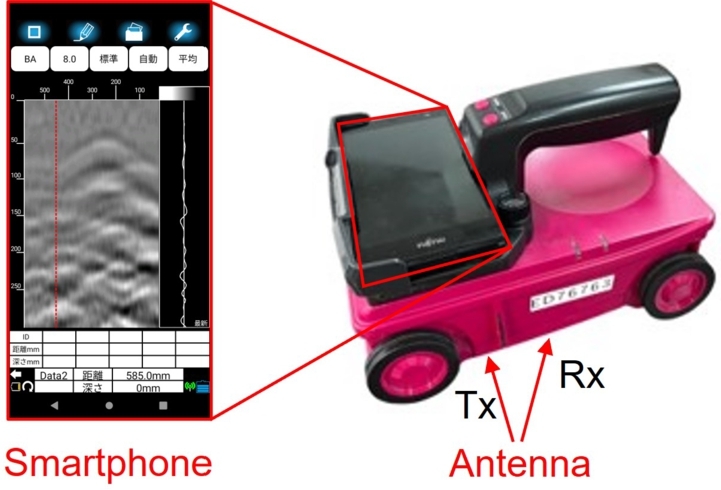
Smartphone-equipped handheld GPR device used in this research.

The state-of-the-art smartphone-equipped handheld GPR device used in this study (Fig. 1) is lightweight, approximately 1 kg, allowing for the equipment to be lifted for measurements. This makes it suitable for inspecting the rear and sides of structures. Data from the GPR device is transmitted wirelessly to a smartphone where it undergoes computation and is displayed in real-time. Inspectors interpret the subsurface structure using a B-scan (Fig. 3), a gray scale image representing the amplitude of the received waveform. The reason inspectors use B-scan is that it is an image obtained by utilizing the property of electromagnetic waves (EMW) reflecting off media with different relative permittivity, making it suitable for understanding the conditions subsurface. The advantage is that it allows for NDI, which can reduce costs and is also suitable for surveying valuable historical structures. Moreover, devices that process and analyze data in real time are widely available, enabling quick decision-making and immediate action on site. Conventionally, this B-scan image is primarily used by inspectors to assess the arrangement of highly reflective rebar. The disadvantage is that it requires inspectors to interpret the images, thus introducing their experience and subjectivity into the process, and damages or other features with low reflectivity become even more challenging to detect accurately. Recent studies have been conducted to detect less reflective damage and connect it to the diagnosis of deterioration [20], [21], [22], [23], [24], [25], [26], [27], [28].

In light of the substantial problem posed by aging infrastructures, there is a pressing need for technology capable of autonomously identifying and locating structural damage, thus reducing reliance on inspectors' skill or experience of inspections. An algorithm that can map the internal condition of infrastructure in a fully automated, real-time, and cost-effective manner, and that is implementable on smartphones, would significantly enhance the efficiency of structural inspections.

To map damage within structures, this study aims to develop a method to estimate the relative permittivity of concrete and the depth of a reflected object with millimeter-order accuracy using a smartphone-equipped GPR. In order for this method to be implemented on a smartphone. However, the data derived from GPR is expressed in terms of the round-trip propagation time, which signifies the duration required for the transmitted EMW to return upon reflecting off an object. The propagation velocity *C* is influenced by the medium's relative permittivity *ϵ*.

(1) $$C=\frac{C_{0}}{\sqrt{\epsilon }}$$

Here, $C_{0}$ represents the speed of light in the air. The speed of EMW propagating in the air is known as the speed of light, $C_{0}$, a fundamental constant in physics. Consequently, the depth of the object *d* can be calculated by,

(2) $$d=\frac{CT}{2}$$

In this equation, *T* denotes the round-trip propagation time of the EMW reflected off an object. Notably, when the medium in question is concrete, its relative permittivity can vary extensively from 4 to 20, contingent on the mix and water content [29]. Using Eq. (1) and Eq. (2), it can be deduced that the estimated depth would be a factor of the $1/\sqrt{5}$ smaller when the relative permittivity is 20 compared to when it is 4. Thus, a reliable relative permittivity is essential to accurately determine the depth of damage within structures and the location of rebar and buried objects.

While the GPR device used in this study allows for the adjustment of relative permittivity, it does not estimate the permittivity itself. Consequently, during measurements, the relative permittivity is set by back-calculating it from known cover thickness of a rebar or known thickness of a wall. However, this approach is only applicable when there is a wall or rebar with a known ‘true’ thickness.

**Figure 2:**
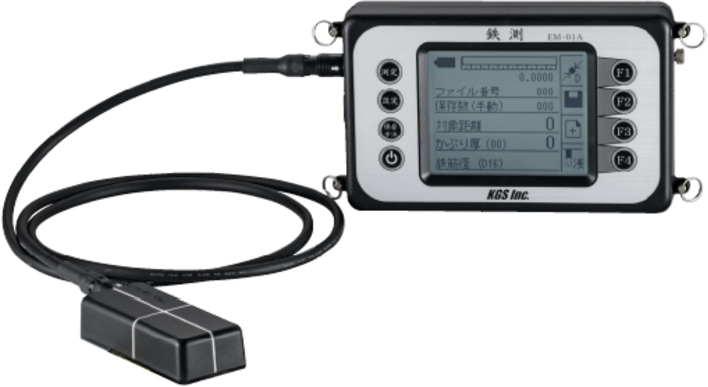
Equipment of EMI method.

Electromagnetic induction (EMI) (Fig. 2) provides an alternative method, allowing for the estimation of cover thickness by measuring the electromagnetic field generated by rebar, followed by back-calculating the relative permittivity. By applying this estimated relative permittivity to all observation points, a depth distribution can be derived. However, utilizing two NDI methods is labor-intensive and inefficient. Additionally, the EMI method is limited to ferromagnetic materials, such as rebar, and has a detection depth limit of approximately 100 to 200 mm, which is shallower than that of GPR. Consequently, while measurements within a depth of 100 mm may suffice for accurately determining rebar cover thickness, a more comprehensive understanding of subsurface conditions requires knowledge of relative permittivity at depths greater than 100 mm. This deeper understanding enables more precise assessments of internal structural conditions.

When estimating the relative permittivity using only GPR, it is typically estimated from the reflection coefficient. The relationship between the reflection coefficient ($\gamma_{ac}$) from the air layer to the concrete surface and the relative permittivity is given by,

(3) $$\gamma_{ac}=\frac{1-\sqrt{\epsilon_{c}}}{1+\sqrt{\epsilon_{c}}}$$

In this model, the relative permittivity of air is assumed to be 1. The actual relative permittivity is determined from the reflection coefficient, which is the ratio of the amplitude of the reflected wave from the concrete surface $A_{ac}$ to the amplitude of the reflected wave in the perfect conductive zone (total reflection, e.g., from a metal plate) $A_{p}$:

(4) $$\gamma_{ac}=\frac{A_{ac}}{A_{p}}$$

Hence, the relative permittivity can be obtained from Eq. (3) and Eq. (4) using the following,

(5) $$\sqrt{\epsilon_{c}}=\frac{A_{p}-A_{ac}}{A_{p}+A_{ac}}$$

Ji-Young Rhee et al. [30] employed the aforementioned method to investigate seasonal variations in permittivity from actual concrete slabs of varying ages on a highway in Korea. However, this method, being capable of estimating the relative permittivity only near the concrete surface and not accounting for depth variation, does not align with the objective of this study, which is to ascertain the depth of the object. Furthermore, Erfansyah Ali et al. [7] posited that to apply this method, maintaining a sufficient height of the antenna from the surface is crucial. This is due to the interference of the received waveform with direct waves, reflections from the concrete surface, and reflections from the rebar when the antenna is proximate to the surface, thereby rendering the separation of $A_{ac}$ and $A_{p}$ infeasible. From this perspective, this method is suited for air coupling and is incompatible with the ground coupling type GPR equipment used in this study, which positions the antenna close to the concrete surface. Efforts to estimate relative permittivity using machine learning techniques, such as deep learning, are underway [31], but these require a large volume of high-quality training data and are not suitable for rigorous and quantitative estimation. Algorithms with less computational load are preferable for real-time operation on smartphones.

**Figure 3:**
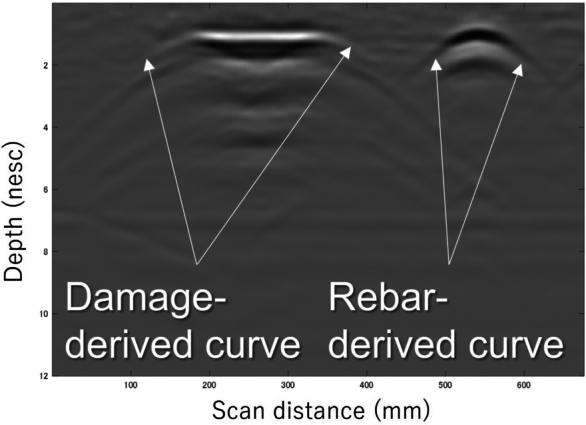
B-scan showing reflection curves generated from damage model and rebar model.

Given that radar captures reflections from buried objects not only directly above but also from in front and behind them, it generates an upward convex hyperbolic reflection waveform ([32]). This waveform, referred to as the reflection curve in this study, deviates from an rigorous hyperbola due to the inter-antenna distance, which is the distance between the transmitting and receiving antennas, the air layer between the antenna and the concrete, and the diameter of the rebar. While the curve primarily appears in rebar and buried pipe, it also emerges in damage and other objects at the boundary of media with different relative permittivity (Fig. 3). Thus, we have elected to leverage this curve in this study, as we believe it can be applied to any reflective object, not just rebar and buried pipes. If we can extract information from every element in the medium and estimate the medium properties, it could lead to a higher resolution of the internal conditions.

The reflection curve can be theoretically derived by calculating the propagation path of the EMW. Subsequently, relative permittivity can be estimated by identifying parameters that yield a theoretical curve closely matching the observed reflection curve. This method is referred to as the curve fitting method throughout this paper. Since the attenuation of the EMW signal with depth, it is essential for the algorithm to be efficient enough to estimate the relative permittivity even in the presence of weak signals, and to do so in a fully automated manner, while minimizing computational load.

Raffaele Persico et al. [33] obtained the shape of the theoretical curve using a fourth-order equation with air and soil as the medium. They realized that estimating relative permittivity from curve fitting is challenging for non-contacting GPR systems. However, the ground coupling type GPR devices addressed in this study show significant changes in the theoretical curve when the parameters of relative permittivity and object depth vary. [34] utilized this property to estimate the position and depth of rebars. Their proposed method concluded that up to a rebar diameter of 32 mm, assuming a rebar radius of zero does not significantly affect the estimated cover thickness. [35] proposed a novel subsurface pipeline mapping and 3D reconstruction method that integrates GPR scanning and camera images. This approach allows for the detection of multiple pipelines and the estimation of their positions and radii, even without perpendicular scanning, under the assumption that the propagation velocity of electromagnetic waves is known. [36] proposed an integrated approach to simultaneously determine the horizontal position, depth, and size of the rebar, estimating depth using curve fitting based on the method of [34]. For this, they identified the zero offset within the GPR scan and eliminated the impact of noise in the GPR signal. O. Klaudia et al. [37] introduces data filtering techniques using wavelet analyses and Gabor filtration to address noise issues affecting the quality of the radargrams, thereby enabling even inexperienced operators to handle the data GPR. The approach is designed to automatically detect buried pipes by dealing with noise problems that impact the quality of the radargrams. I. Giannakis et al. [38] points out that conventional hyperbola fitting is based on the assumption that the investigated medium is a homogeneous half-space, and that the target is an ideal reflector with zero radius. Through a series of synthetic experiments and laboratory experiments, they demonstrate that for practical GPR survey, hyperbola fitting is not suitable for simultaneously estimating both the velocity of the medium and the size of the target, due to its inherent nonuniqueness, making the results unreliable and sensitive to noise. In these previous studies, the focus has predominantly been on the detection of hyperbolic curves alone, and even among research aimed at estimating depth, discussions concerning a few percent of error are scarce.

Moreover, various studies have focused on error percentages. Xie et al. ([39][40]) developed error propagation models for hyperbolic fitting both before and after measurement, comparing traditional fitting algorithms (e.g., standard hyperbola fitting [41]) with more advanced CLS-based methods [42], discussing their respective error rates. Additionally, optimization-based fitting methods ([38], [43], [44]) have been proposed, offering potential improvements in convenience and accuracy. In this study, rather than concentrating on the influence of parameters on error rates, we emphasize practical application. We propose an algorithm designed to estimate depth fully automatically at the accuracy level required by practitioners, while excluding the influence of object size. Some of the previous studies have considered antenna spacing in their models, but they only adopt the geometric antenna distance and do not account for the actual electromagnetic wave propagation path.

In this study, we employ the curve fitting method to estimate relative permittivity and depth, as we believe that a ground coupling type GPR device can accurately estimate the relative permittivity through curve fitting between the theoretical curve and the observed reflection curve. In this context, our objective is to meet the accuracy standards prescribed by the Japanese Society for Non-Destructive Inspection (JSNDI) for the EMI method using our proposed technique. The stipulated performance criterion is within ±5% for depths exceeding 40 mm. Accordingly, in this study, we aim to achieve estimation accuracy with an error margin of less than 5%.

• We propose an innovative method for depth determination that does not require consideration of the buried object's size. This approach involves dividing the observed hyperbolic curve into left and right sections and conducting curve fitting separately for each side. Importantly, we exclude the area near the vertex of the theoretical hyperbolic curve from the fitting process. This technique allows for a more accurate estimation of depth by minimizing the influence of the object's shape and ensuring that the fitting process is tailored to the specific characteristics of each curve section.
• We successfully automated the extraction of hyperbolic curves from B-scan by focusing on the bilateral symmetry of the hyperbolas. This was achieved even at depths of 180 mm, which is considerable for the intended purpose of this device. Additionally, we successfully determined the central line of the hyperbola, which is crucial for curve fitting. All these calculations were made possible in approximately 0.075 seconds.
• By optimizing the inter-antenna distance, which reflects the actual propagation path of the EMW, we were able to estimate the depth of the rebar with a maximum error of 2.67%. Notably, this accuracy is independent of depth.

The structure of this paper is as follows: Section 2 provides an overview of the GPR equipment used in this study and the experimental data. Section 3 details the proposed method using curve fitting techniques with a rigorously optimized inter-antenna distance. The paper concludes with Section 4.

## 2 Experimental framework and data acquisition

### 2.1 GPR equipment

**Figure 4:**
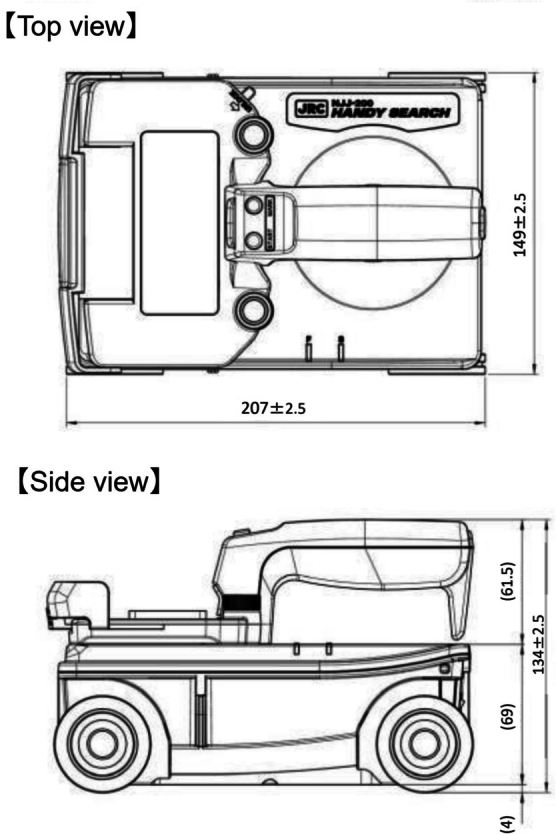
External view of specimen.

The GPR device used in this study is illustrated in Fig. 1, and its specifications are outlined in Table 1. This GPR device is notably lightweight and compact, with dimensions of 149 mm (width) × 207 mm (height) and a weight of approximately 1 kg (Fig. 4). LED lights are equipped on the front and sides of the GPR body, which project lines onto the concrete surface when lit, aiding in aligning the GPR's position. The smartphone and the sensor body communicate via wireless LAN. Utilizing a smartphone as the display allows for the use of the latest CPU simply by replacing the smartphone. The radar system's transmission wave used in this study is of an impulse system, utilizing an antenna with a center frequency of 2100 MHz within the bandwidth of 700 MHz to 3500 MHz.

**Table 1:** Specifications of the radar.

Transmitting and Receiving Antenna	Separated (Bi-static)
Radar System	Pulse Radar
Frequency Band	700 MHz-3500 MHz
Measurable depth	450 mm (For concrete with relative permittivity 6.2)
Horizontal distance resolution	2.5 mm
Maximum scanning speed	Approx. 80 cm/s
Data communication between	Wireless LAN
GPR and smartphone

### 2.2 Computational environment

Table 2 shows details of our computational environment and software used to implement our proposed method. We used MATLAB for implementing all the functionalities involved in our proposed method. MATLAB toolboxes used for this purpose are – MATLAB, Image Processing Toolbox, Signal Processing Toolbox and Statistics and Machine Learning Toolbox.

**Table 2:** Computational Environment and Software.

Item	Specifications
Operating System	Windows 10
CPU	Intel(R) Core(TM) i9
Memory	32 GB
MATLAB version	R2023a

### 2.3 Data collection

While the generation of curves can be influenced by reflections from various objects, for this data collection, a thin, elongated piece of metal was utilized as a rebar model.

**Figure 5:**
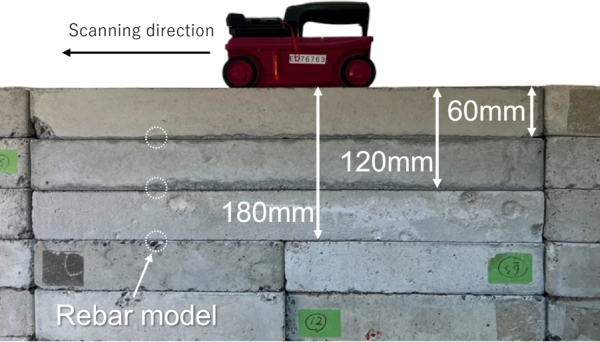
External view of specimen.

**Figure 6:**
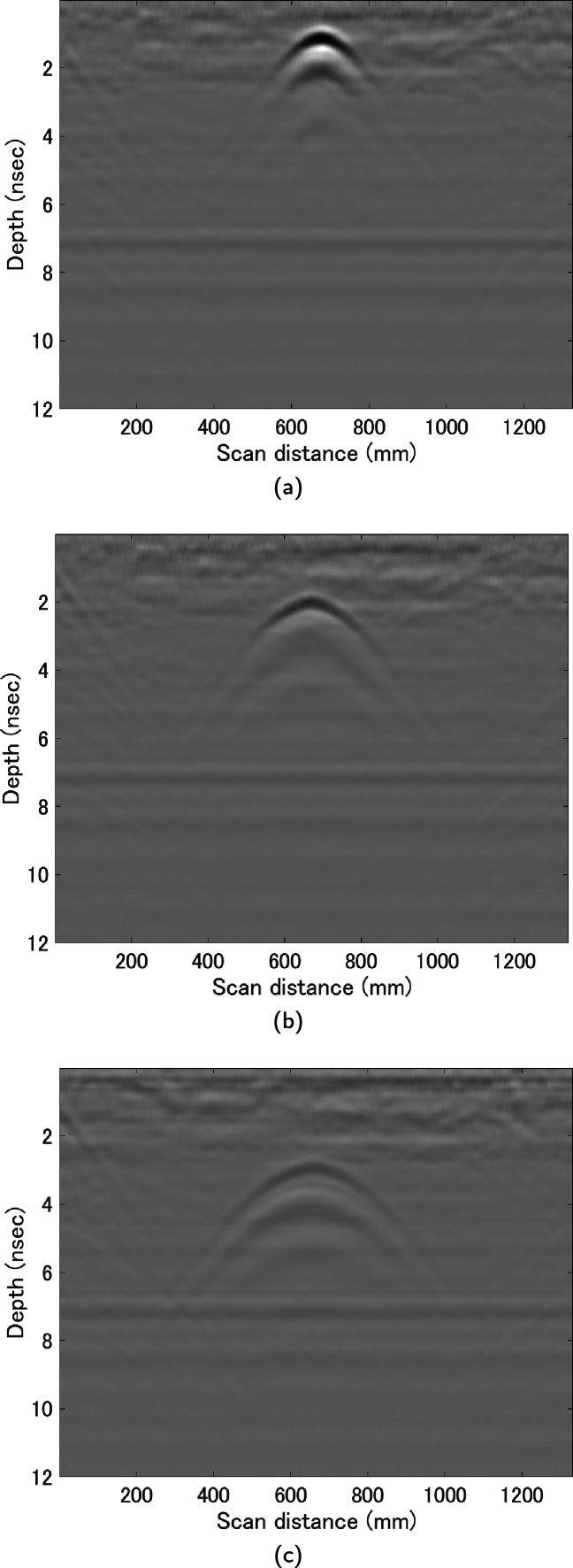
Example of B-scan observed in the experiment: Depth of rebar model (a) 60 mm, (b) 120 mm, (c) 180 mm.

In order to develop the algorithm, data was gathered using concrete blocks of two different dimensions (300 mm × 300 mm × 60 mm, 600 mm × 300 mm × 60 mm) which were stacked in combinations without rebar. A rebar model serving as a reflective object was positioned between the concrete blocks, with measurements taken at three distinct depths of 60 mm, 120 mm, and 180 mm, each repeated five times. Fig. 5 presents an external view of the specimen. The diminishing intensity of the color from 60 mm to 180 mm depth indicates a decrease in amplitude (Fig. 6). The B-scans in this study consider the first peak as the time zero. Consequently, all these B-scans are treated as received waveforms within the concrete. This suggests that as the depth increases, the signal-to-noise ratio (S/N) decreases, making it more challenging to automatically extract the hyperbolic curves and perform fitting.

## 3 Proposed curve fitting method

In this chapter, we describe the proposed depth estimation in concrete method. The chapter outlines the step-by-step procedure for performing curve fitting. The proposed workflow is divided into two major phases:

Phase 1: Fully automatic detection of the centerline of reflection curves.
Phase 2: Hyperbolic curve fitting based on the centerline defined by Phase 1.

In Phase 1, we address the identification of the centerline passing through the vertex on the hyperbola, which is necessary for curve fitting. The theoretical curves to be fitted are fixed within a narrow range around this centerline. This approach prevents the output of theoretically derived curves that are clearly misaligned with the center. Moreover, because the theoretical curves are fitted along a line rather than across an area, it significantly reduces the computational load.

In the following section, after discussing the method for generating theoretical curves, we will detail Phases 1 and 2 respectively. Furthermore, we will elaborate on the model that considers the inter-antenna distance, which is crucial for achieving millimeter-order high-precision estimation.

### 3.1 Generate a theoretical curve

In generating the theoretical curve, the propagation path is modeled. The GPR device discussed in this study is a ground coupling type, and the distance from the antenna to the concrete surface is 4 mm (Table 1). This value is significantly small compared to the wavelength *λ* in air of 142.9 mm for the center frequency of 2100 MHz of this antenna (Eq. (6)), and the air layer is thus ignored here.

(6) $$\lambda =\frac{3\times 10^{11}\text{[mm/s]}}{2100\times 10^{6}[\text{Hz}]}=142.9\text{[mm]}$$

**Figure 7:**
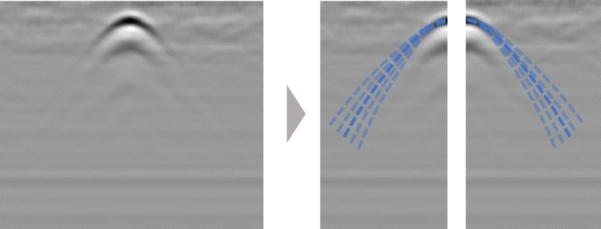
Curve fitting is performed by dividing the extracted hyperbolic curve into left and right sides using the vertex as the boundary.

In this study, curve fitting is conducted by isolating each side of the curve at the vertex, focusing solely on the skirt of a single hyperbolic curve (Fig. 7). This approach not only enables the generation of theoretical curves without the need to know the diameter of the rebar but also facilitates the fitting of curves originating from one endpoint for objects of various shapes, including damage.

**Figure 8:**
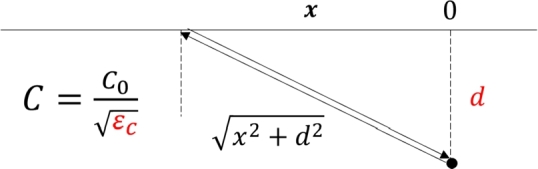
Model of EMW propagation in concrete (Red letters are variables). *x*: the horizontal distance from the center of the rebar, *d*: vertical depth of the rebar center, *ϵ*_*c*_: relative permittivity of concrete, *C*: light velocity in concrete. *C*_0_: light velocity in the air.

Fig. 8 shows a basic propagation model that does not consider the inter-antenna distance. Once the depth and permittivity of the rebar model are established, the theoretical curve can be uniquely defined. Initially, without considering the inter-antenna distance, the equation of the theoretical curve $t_{0}$ is,

(7) $$t_{0}(x)=2\frac{\sqrt{x^{2}+d^{2}}\sqrt{\epsilon_{c}}}{C_{0}}$$

where *x* is the horizontal distance from the center of the rebar, *d* is the vertical depth of the rebar center, $\epsilon_{c}$ is the relative permittivity of the concrete, and $C_{0}$ is the speed of light in the air. As the theoretical curve represents a round-trip propagation time, the one-way propagation time is doubled.

**Figure 9:**
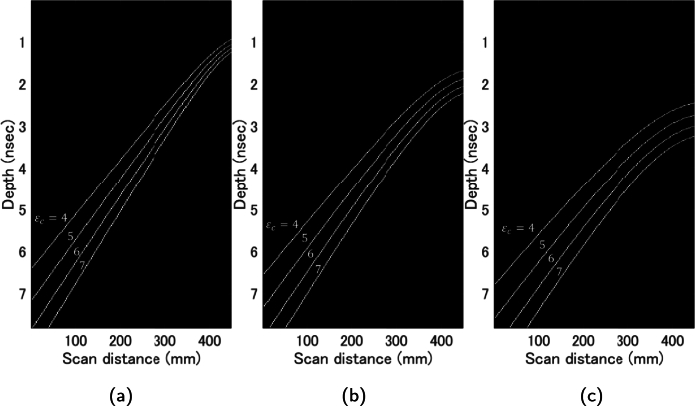
Theoretical curves for various permittivities at the same depth. Depth of rebar model: (a) 60 mm, (b) 120 mm, (c) 180 mm, Relative permittivity 4, 5, 6, 7.

Fig. 9 shows a superimposed drawing of a typical theoretical curve generated by changing the rebar depth *d* and relative permittivity $\epsilon_{c}$. The theoretical curves vary in this manner.

### 3.2 Detect fully automatic center line

In order to achieve accurate estimation, it is imperative to fully automate the extraction of the fitting range. It is crucial to specify as narrow a range as possible during the extraction stage in order to remain unaffected by noise. In particular, it is important to identify at what depth the curve response is located because radar attenuates as it goes deeper into the medium.

Initially, the automation of center line alignment is necessary. Curves generated from buried pipes and rebar are symmetrical. Considering that the curve originating from the rebar is symmetrical on both sides, we focus on the symmetry of the curve. By defining a line in a certain depth direction and folding over a specific interval around that line, the symmetry at the curve's center becomes significantly stronger. Symmetry is calculated using an image of the observed waveform with edge detection. This is because the left-right symmetry of a received waveform, which decays in the depth direction, highly depends on the symmetry of the waveform's shallow part when calculated as is. However, as the edge-detected image is binary data, the information is uniform regardless of the depth direction. Edge detection is performed using the Canny method [45]. Unlike other edge detection methods, the Canny method uses two different thresholds and includes in the output only weak edges that are connected to strong edges. Therefore, it is less susceptible to noise compared to other methods, increasing the likelihood of accurately detecting weak edges and yielding a stable output image. The threshold is determined based on experience. The center line is identified using prominence. Based on our hypothesis, when the center line is positioned at the vertex of the hyperbola, an abrupt enhancement in symmetry is observed. We used prominence as a tool to identify the hyperbola's center line. The ‘prominence’ of a peak measures how much the peak stands out due to its intrinsic height and its relative location to other peaks. A peak that is isolated at a lower position can have a higher degree of prominence than a peak at a higher position but not standing out from its surroundings. A threshold for prominence is determined empirically as half or more of the maximum value indicating symmetry, and this is extracted as the first candidate for the curve's center line.

**Figure 10:**
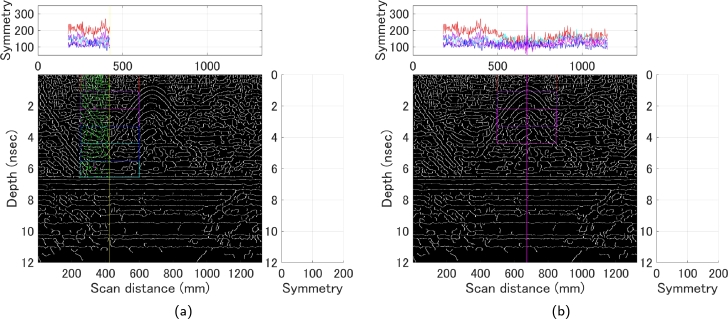
Horizontal search; (a) Symmetry strength in several depth zones (b) Candidate center lines extracted because of Fig. 9 (a), illustrated using a 60 mm B-scan as an example.

**Figure 11:**
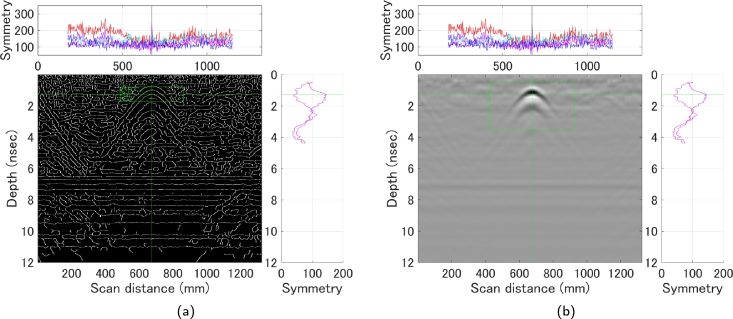
Vertical search; (a) Results of a search in the depth direction based on the candidate center lines obtained in Fig. 10 (b) Final extracted fitting range, illustrated using a 60 mm B-scan as an example.

In addition, by fixing the candidate center line searched from the horizontal direction and searching for this symmetry in a narrower range, this time in the depth direction, it is possible to determine up to the depth zone where the curve response is located. In fact, by calculating the symmetry in several depth zones at the time of the horizontal search (Fig. 10), the curve response could be extracted more robustly, since some depth zones could be known at the time of the first stage of search (Fig. 11). This method allows for the fully automated extraction of the curve response even with a very weak signal of 180 mm. Using the computational environment listed in Table 2, the computational costs for identifying hyperbolas and determining their center lines are summarized in Table 4. In our proposed measurement method, the computation time depends on the number of center line candidates extracted during the horizontal search, although the difference is not significant, with all data requiring the same computation time.

### 3.3 Hyperbolic perform curve fitting

The detected center line serves as a guideline upon which hyperbolic curve fitting is performed. Extrema in both the time direction and the horizontal direction of the received image are extracted (Fig. 13). The least squares between these points and the theoretical curve are computed for each reception point, and the theoretical curve with the smallest total is taken as the solution.

**Figure 12:**
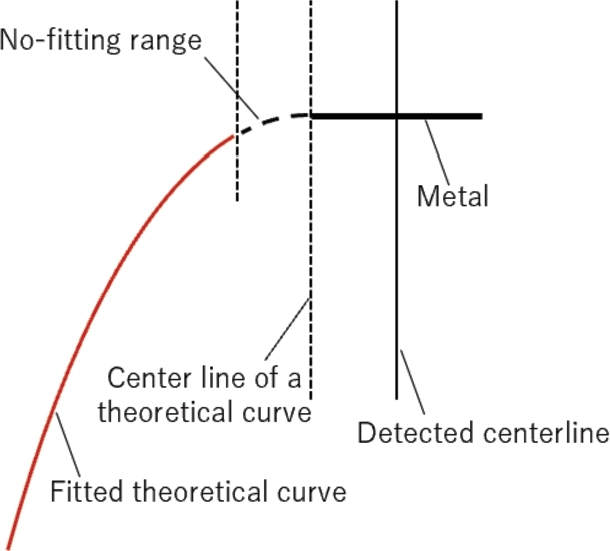
Actual fitting area and spatial relationship.

Fitting is conducted separately for the right and left sides. Furthermore, as shown in Fig. 12, one millimeter from the center of the theoretical curve is not used for fitting. This is because we believe that the area near the vertex of the curve is influenced by the shape of objects, such as the diameter of rebar. By conducting fitting separately for each side and excluding the area near the vertex of the theoretical curve, it becomes unnecessary to consider the specific shape of objects. However, under the premise that the estimated depth remains consistent, the theoretical curve with the smallest cumulative least squares is selected. Consequently, while the relative permittivity may vary for each side, the depth remains consistent, resulting in the same hyperbolic curve being the solution for each side's fitting. As illustrated in Fig. 12, it is important to note that the rebar model has a width, and thus the center line of the fitted hyperbola does not necessarily coincide with it.

**Figure 13:**
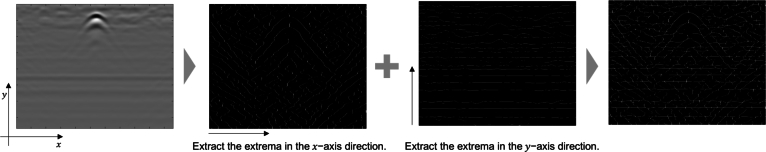
Method for binarization of data for curve fitting.

**Figure 14:**
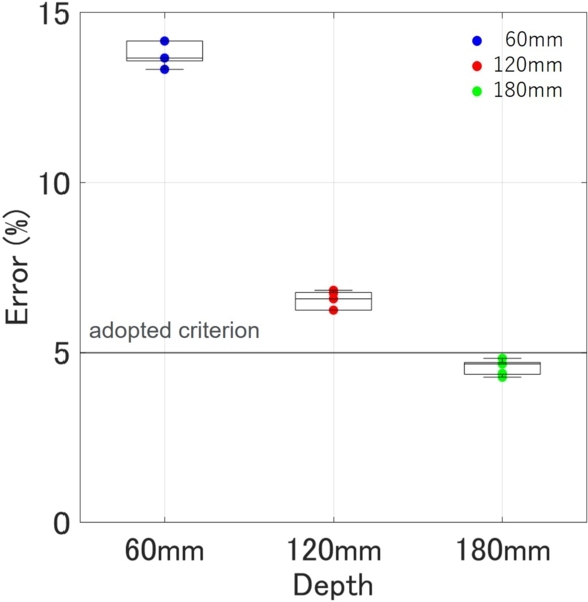
Relationship between actual depth and error of estimated depth.

The results are shown in the Fig. 14. The goal was to achieve 5%, but the accuracy was poor, with errors exceeding 5% except for 180 mm. A notable characteristic is that the error increases as the depth becomes shallower. From this observation, it is inferred that as the depth becomes more shallow, it is imperative to incorporate elements that have a greater impact into the theoretical curve.

### 3.4 Accounting for the inter-antenna distance effect

To generate a more accurate theoretical curve, we considered the inter-antenna distance, which is the distance between the transmitting and receiving antennas. These antennas use different units and are spatially separated from each other. The geometrical inter-antenna distance spans several tens of millimeters, a non-negligible factor when estimating depths from 60 mm to 180 mm. However, discerning the actual propagation path of EMW proves to be extremely challenging.

**Figure 15:**
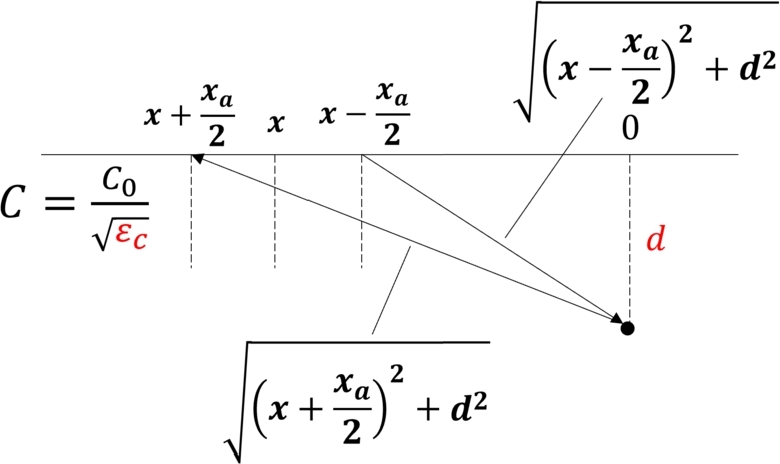
Exact propagation path model taking into account the inter-antenna distance (Red letters are variables). *x*: position of center of antenna from the rebar, *x*_*a*_: distance of inter-antenna, *d*: vertical depth of the rebar center, *ϵ*_*c*_: relative permittivity of concrete, *C*: light velocity in concrete. *C*_0_: light velocity in the air.

To address this, we incrementally widened the theoretical curve's inter-antenna distance from 0 mm and analyzed the resulting estimations to determine the appropriate distance. The least squares method was employed for fitting, with depth estimations made at 0.1 mm intervals and relative permittivity estimated in increments of 0.01. The figure presents the estimation results as the inter-antenna distance is expanded by 1 mm. The theoretical curve is constructed based on Fig. 15 of the model, using Eq. (8).

(8) $$t(x)=\frac{\sqrt{{\left(x-\frac{x_{a}}{2}\right)}^{2}+d^{2}}\sqrt{\epsilon_{c}}}{C_{0}}+\frac{\sqrt{{\left(x+\frac{x_{a}}{2}\right)}^{2}+d^{2}}\sqrt{\epsilon_{c}}}{C_{0}}=\frac{1}{2}(t_{0}(x-\frac{x_{a}}{2})+t_{0}(x+\frac{x_{a}}{2}))$$

It's important to note that we disregarded the gap between the antennas and the concrete surface in our model. The GPR used in our study has an approximate gap of 4 mm (Fig. 4). As shown in the table, this radar's sampling frequency is 64 GHz, resulting in a distance of 46.9 mm per sample in air. The 4 mm gap cannot be captured by this radar, and the EMW have already penetrated the concrete from the first point after being transmitted. Therefore, we concluded that there is no need for a zero offset, and this gap was not considered in the creation of our theoretical curve model.

**Figure 16:**
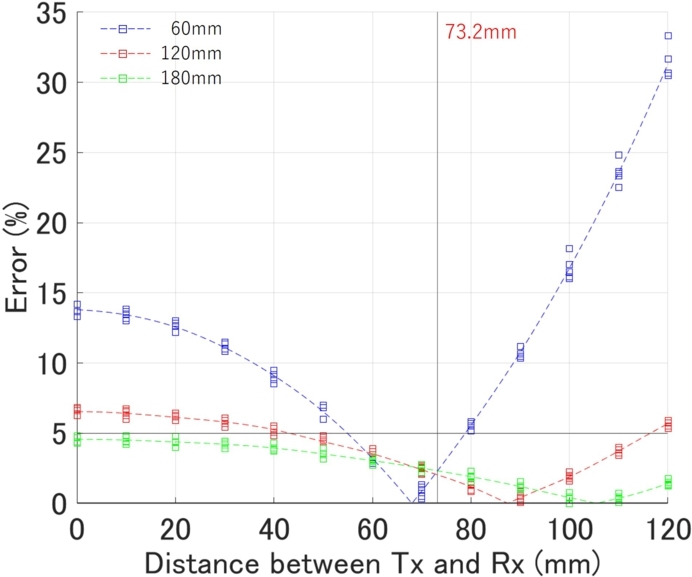
Relationship between inter-antenna distance and error of estimated depth.

Fig. 16 presents the error results for varying inter-antenna distances at depths of 60 mm, 120 mm, and 180 mm, respectively. The zero-crossing points correspond to the theoretical inter-antenna distance. In practice, due to inherent uncertainties, there is a slight deviation from this value depending on the depth. Given our research objective of achieving an error rate of less than 5%, we have adopted an inter-antenna distance of 73.2 mm, which offers the smallest maximum error, for the generation of the theoretical curve.

In curve fitting, depth and relative permittivity are the variables, and these two values are estimated simultaneously. The accuracy is determined by the depth for which the true value is known.

**Figure 17:**
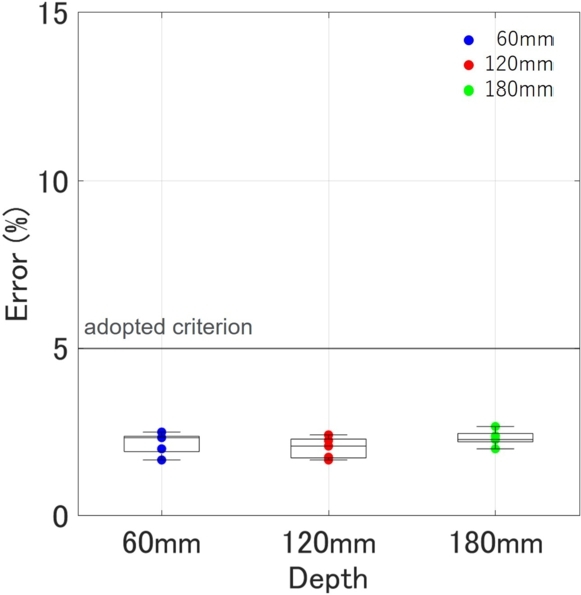
Relationship between actual depth and error of estimated depth by final fully-automatically algorithm for *x*_*a*_ = 73.2 (mm).

**Figure 18:**
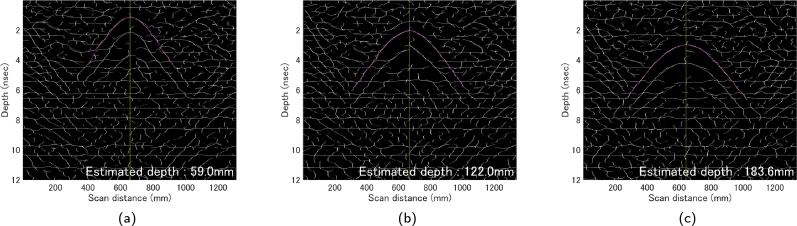
Result of curve fitting; (a) 60 mm, (b) 120 mm, (c) 180 mm.

The results of the final algorithm for depth estimation are presented in Table 3, and the outcomes are plotted in Fig. 17. An example of curve fitting for each depth is shown in Fig. 18. The proposed method estimates the relative permittivity and depth simultaneously. The only constraint is that the estimated depth remains the same for both sides, hence, the estimated relative permittivity varies between the sides. Looking at the estimated depth results in Fig. 17, the maximum error is 2.67%, and all fall within a 5% error range. The required performance of the inspection equipment, as specified in the ‘Method for detecting rebars in concrete structures by EMI method’ (NDIS3430) by the JSNDI, necessitates that the measurement accuracy of the cover thickness be within ±5% at a depth of 40 mm or more. Fig. 17 demonstrates that the accuracies of all data meet this requirement. Furthermore, NDIS3430 requires that the maximum range of detectable depth be at least 100 mm, and the results for 120 mm and 180 mm are within ±5% of the required accuracy for the EMI method. Although in reality, a cover thickness of 180 mm is extremely rare, the fact that the accuracy of the proposed method is not influenced by depth, unlike the EMI method, which significantly loses accuracy beyond 100 mm, demonstrates the robustness of this method and is noteworthy.

**Table 3:** Final result of proposed method. Depth (*d*) and permittivity (*ϵ*_*L*_ and *ϵ*_*R*_) are estimated simultaneously. The calculation time is the total time taken from loading the data to outputting the results.

Actual depth (mm)		Estimated $\epsilon_{L}$ and $\epsilon_{R}$ and *d* (mm)			Relative error (%)
60	#1	6.47	6.29	58.5	2.50
	#2	6.25	6.23	59.0	1.67
	#3	6.31	6.46	58.6	2.33
	#4	6.39	6.41	58.6	2.33
	#5	6.39	6.26	58.8	2.00
	Avg.	6.36	6.33	58.7	2.17

120	#1	5.97	6.01	122.0	1.67
	#2	6.03	5.91	122.9	2.42
	#3	5.97	5.88	122.5	2.08
	#4	5.98	6.03	122.1	1.75
	#5	6.05	5.86	122.7	2.25
	Avg.	6.00	5.94	122.4	2.03

180	#1	5.72	5.70	184.8	2.67
	#2	5.79	5.71	184.1	2.28
	#3	5.77	5.74	184.3	2.39
	#4	5.81	5.82	183.6	2.00
	#5	5.82	5.75	184.1	2.28
	Avg.	5.78	5.74	184.2	2.32

In the computational environment described in Table 2, the total time taken from loading the received images to estimating the depth is summarized in Table 4. We can say that our proposed automated estimation method can calculate depth with a computational time with approximately 5 seconds or less for any depth. Compared to conventional manual measurements using the two methods of GPR and EMI, this performance is a remarkable improvement.

**Table 4:** Computational costs of Phase 1 (left column) and the total proposed algorithm (right column).

Actual depth (mm)	Calculation time of Phase1 (left column) and Total (right column) (Secs.)											
Data number	#1		#2		#3		#4		#5		Avg.	
60	0.082	4.63	0.073	3.85	0.075	3.83	0.074	3.87	0.071	3.74	0.075	3.98
120	0.080	4.18	0.072	4.19	0.082	4.35	0.075	4.24	0.073	4.24	0.076	4.24
180	0.079	4.40	0.075	4.43	0.075	4.39	0.072	4.45	0.071	4.41	0.075	4.42

## 4 Conclusion

The aim of this research was to precisely estimate the depth of concrete and reflected objects with millimeter-level accuracy, thereby enabling the accurate mapping of detected damage and reflected objects. We concentrated on the reflection curves generated by object reflections and undertook high-precision estimation by conducting curve fitting with a rigorously derived theoretical curve, which took into account the inter-antenna distance. The key conclusions drawn from this research can be summarized as follows:

• We automated the process of extracting hyperbolic curves simply by inputting the received image. This was achieved by focusing on the bilateral symmetry of the hyperbolas. The method proved effective even at considerable depths of 180 mm, aligning with the intended purpose of this device. Additionally, we successfully pinpointed the central line of the hyperbola, which is critical for curve fitting. All these calculations were completed in approximately 0.075 seconds.
• We proposed a method for determining depth without considering the size of the buried object by dividing the observed hyperbolic curve into left and right sections and excluding the area near the vertex of the theoretical hyperbolic curve from the fitting process. This method holds promise for future application, not only to rebar or buried pipes but also to other objects such as voids and aggregates, provided that a unidirectional curve can be obtained from irregularly shaped objects.
• By optimizing the inter-antenna distance to reflect the actual propagation path of EMW, we were able to estimate the depth of rebar with a maximum error of 2.67%. Notably, this accuracy is independent of depth, making it comparable to the EMI method and nearly meeting the performance requirements set by the JSNDI. The consideration of inter-antenna distance is crucial when creating a theoretical hyperbolic curve.

Currently, the inter-antenna distance cannot be set automatically and requires manual adjustment. In our view, the value of the inter-antenna distance is independent of the medium or environment in which it is utilized. If future investigations confirm that the inter-antenna distance is unaffected by various mediums or environments, then this effective inter-antenna distance will become a unique value specific to each device. By pre-measuring and setting the effective inter-antenna distance for each device, the algorithm can be used repeatedly and indefinitely, thereby enhancing its versatility. Additionally, if the relationship between the effective and geometric inter-antenna distances, as well as the behavior of electromagnetic waves, is elucidated in future studies, it may become feasible to automatically determine the effective inter-antenna distance.

To extend the applicability of this approach to reflective waveforms that mimic real-world conditions, further validation is necessary. This study utilized a piece of metal as the reflective object; however, validating the methodology with actual reflective objects encountered in real scenarios, such as structures damaged or objects originating from rebar, is essential for future research. Moreover, enabling automatic setting of the inter-antenna distance could significantly increase the technology's general applicability and practicality. Therefore, the future outlook of this research includes optimizing the inter-antenna distance and expanding the application to reflective waveforms resembling actual conditions.

## CRediT authorship contribution statement

**Shunsuke Iwai:** Writing – review & editing, Writing – original draft, Visualization, Validation, Formal analysis, Software, Resources, Methodology, Investigation, Data curation, Conceptualization. **Tsukasa Mizutani:** Supervision, Project administration, Funding acquisition, Conceptualization.

## Declaration of Competing Interest

The authors declare that they have no known competing financial interests or personal relationships that could have appeared to influence the work reported in this paper.

## Data Availability

The authors do not have permission to share data.
